# Supplementary material for: Immune Checkpoint Expression on Immune Cells of HNSCC Patients and Modulation by Chemo- and Immunotherapy
Source: Int J Mol Sci. 2020 Jul 22;21(15):5181. doi: 10.3390/ijms21155181 (PMC7432918; doi:10.3390/ijms21155181)
Supplement: Supplementary file 1 [file ijms-21-05181-s001.pdf]

Immune Checkpoint Expression on Immune Cells of HNSCC Patients and Modulation by Chemo- and Immunotherapy

Lisa K. Puntigam, Sandra S. Jeske, Marlies Götz, Jochen Greiner, Simon Laban, Marie-Nicole Theodoraki, Johannes Doescher, Stephanie E. Weissinger, Cornelia Brunner, Thomas K. Hoffmann and Patrick J. Schuler

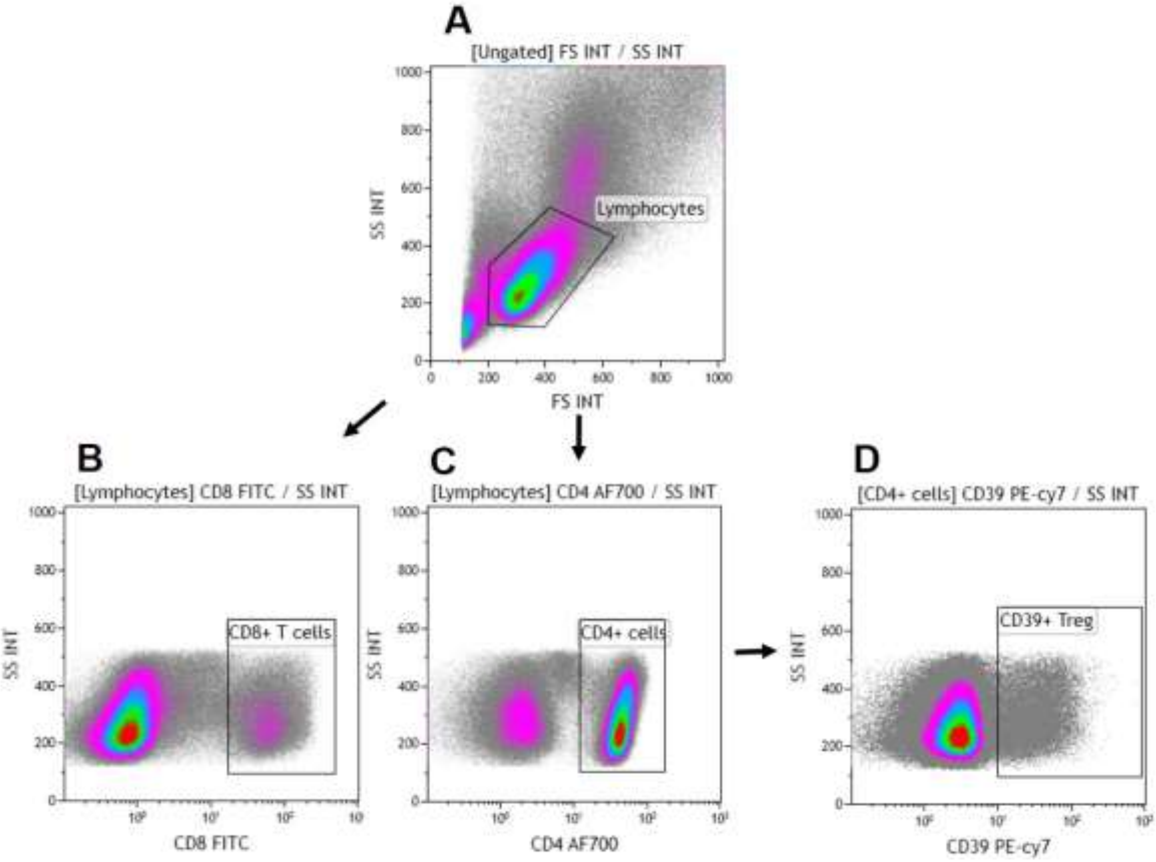

Figure S1. Gating strategy for the identification of T cell subpopulations. The lymphocyte population was gated according to the characteristic size of the lymphocyte fraction with forward and sideward scatter. Cytotoxic T cells were defined as CD8+ cells. T helper cells were defined as CD4+ cells and Treg as CD4+CD39+ cells according to previous literature that identified this subgroup as a highly immunosuppressive subpopulation [36]. Representative density plots are shown.

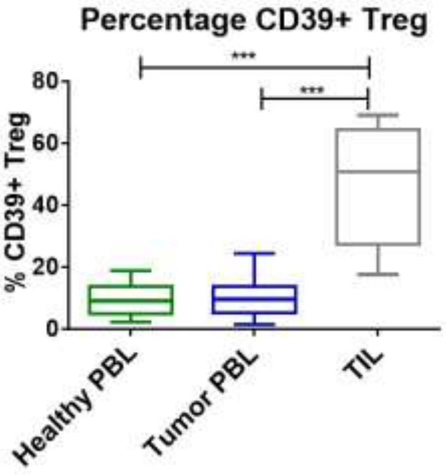

Figure S2. Increased CD39+ Treg frequency in tumor tissue compared to PBL. PBL of 23 healthy donors, 23 HNSCC patients and TIL of 12 of the tumor patients were isolated and the frequency of CD4+CD39+ regulatory T cells was detected by flow cytometry. TIL showed a significantly increased Treg frequency compared to PBL of healthy donors and HNSCC patients. *p*-values < 0.001 (***), *p*-values < 0.0001 (****).
